# Implication of FDG-PET/CT without synchronous colonic lesion in patients with stenotic left-sided colorectal cancer

**DOI:** 10.1038/s41598-021-94030-w

**Published:** 2021-07-19

**Authors:** Jong Il Lee, Sang Sik Cho, Ui Sup Shin, Byong Ho Jeon, Sun Mi Moon, Younjoo Kim, Ki Young Yang, Byung Il Kim

**Affiliations:** 1 Department of Surgery, Korea Cancer Center Hospital, Korea Institute of Radiological and Medical Sciences, 75 Nowon-gil, Nowon-gu, Seoul, Korea; 2 Department of Gastroenterology, Korea Cancer Center Hospital, Korea Institute of Radiological and Medical Sciences, 75 Nowon-gil, Nowon-gu, Seoul, Korea; 3 Department of Nuclear Medicine, Korea Cancer Center Hospital, Korea Institute of Radiological and Medical Sciences, 75 Nowon-gil, Nowon-gu, Seoul, Korea

**Keywords:** Diseases, Gastroenterology, Oncology

## Abstract

Although 18-fluoro-2-deoxy-glucose positron emission tomography/computed tomography (18F-FDG PET/CT) is useful for detecting synchronous colorectal cancer (CRC) in stenotic CRC, long-term outcomes of patients without synchronous FDG-avid lesions are not well reported. We investigated postoperative colonoscopy results in patients with left-sided stenosing CRC without synchronous FDG-avid lesions. In this retrospective review, 754 patients with left-sided CRC without synchronous FDG-avid lesions on preoperative 18F-FDG PET/CT were divided into two groups based on the completeness of preoperative colonoscopy. Propensity score matching was performed to balance baseline characteristics. Results of postoperative colonoscopy were compared in both the unmatched and matched cohorts. At 1 and 5 years after surgery, the cumulative risk of advanced adenoma (AA) or carcinoma (CA) in all patients, risk of CA, and additional surgical risk were 1.8% and 10.1%, 0.1% and 0.4%, and 0% and 0.5%, respectively. In both cohorts, the AA risk was significantly higher in the incomplete colonoscopy group. However, the risk of CA showed no between-group difference in the matched cohort. Additional surgical risk did not differ between the two groups. Thus, the finding of negative FDG-avid lesions in the proximal colon in addition to the target CRC ensures the absence of additional lesions warranting surgical plan changes.

## Introduction

Although the incidence of synchronous colorectal cancer (CRC) is relatively low (1.1–8.1%)^1^, the risk of concurrent colorectal neoplasms, which may require additional surgery on the proximal colon in the case of left-sided CRC through which colonoscopy cannot pass, remains a concern. High-quality perioperative clearing—especially preoperative clearing—has been emphasized to detect and remove synchronous colon neoplasms. However, current guidelines state that colonoscopy should be considered 3–6 months after surgical resection of the obstructive lesions in occlusive cases^2,3^. Preoperative computed tomography (CT) colonography^4^ or double-contrast barium enema have been recommended as alternative interventions, but this method is also impossible in the presence of complete obstruction. Colonoscopy may also be considered after inserting a self-expanding metallic stent or after a decompressive stoma, but this is not possible in all cases. Intraoperative colonoscopy may also be an option; however, colonoscopy during surgery is not easy in this era of laparoscopic surgery. Consequently, if high-quality preoperative colonoscopy does not achieve clearing of synchronous colon neoplasms, the possibility of additional surgery due to the remaining synchronous lesion(s) remains at the time of surgery for index CRC.

18-fluoro-2-deoxy-glucose (18-FDG) positron emission tomography (PET)/CT is a useful modality for staging and detecting recurrences in various malignancies, including CRC^5–8^. Moreover, for many years now, studies have suggested that incidental colorectal adenomas and cancers may be FDG-avid in PET studies^9,10^^.^ Furthermore, 18F-FDG PET/CT has been reported useful for the detection of proximal synchronous lesions in patients with obstructive colorectal cancer^11,12^^.^ 18F-FDG PET/CT has shown high sensitivity and negative predictive value for detecting proximal synchronous CRC in patients with obstructive CRC, enabling negative findings in the proximal colon on PET/CT to exclude proximal synchronous CRC definitively^11^. However, in many cases, significant tumours, especially advanced adenomas, may be missed^13,14^. In addition, very few reports on patients’ actual long-term postoperative colonoscopy results without synchronous FDG-avid colonic lesions exist. Thus, it is still unclear how many of these significant proximal s missed by PET/CT in occlusive CRC cases require additional surgery.

In this study, we investigated the risk of advanced adenoma, adenocarcinoma, and the risk of additional surgery due to these lesions by reviewing the results of postoperative surveillance colonoscopy in patients who underwent surgical resection for left-sided CRC without additional FDG-avid lesions at the preoperative unobservable proximal colon by colonoscopy. We also compared this risk with that of patients who underwent a complete preoperative colonoscopy.

## Methods

### Ethics

This study was approved by the Ethics Committee of the Korea Cancer Center Hospital (approval no. KIRAMS-2021-03-007). All studies were performed in accordance with the approved guidelines and regulations of the institution. The requirement for informed consent was waived by Korea Cancer Center Hospital Institutional Review Board given the retrospective nature of the study.

### Patients

From February 2009 to December 2017, we retrospectively selected patients from the institutional CRC database. The patients included those who underwent primary tumour resection due to pathologically proven adenocarcinoma (CA) with available preoperative PET/CT and pre-and postoperative colonoscopy images. In addition, we excluded patients with Lynch syndrome or other polyposis syndromes. We also excluded patients who underwent total colectomy or right hemicolectomy during which all of the proximal colons were removed. Finally, we excluded patients with any additional FDG-avid colonic lesions in the proximal colon. Thus, 754 patients were selected, and according to the completeness of preoperative colonoscopy, they were divided into two groups: complete (n = 616) and incomplete (n = 138) colonoscopy.

### Postoperative surveillance colonoscopy

All postoperative surveillance colonoscopies were performed by board-certified gastroenterologists at our institution. The total number of colonoscopies performed after surgery was 1435, and the total number of sessions for detecting any adenoma types was 451. Thus, the adenoma detection rate at our institution was 31.4%. The median period of initial and last colonoscopy after surgery was 14 months (interquartile range [IQR], 12–21 months) and 45 months (IQR, 18–60 months). The median number of postoperative colonoscopies per patient was 2 (IQR, 1–2). Advanced adenoma (AA) was defined as any adenoma with a villous component, high-grade dysplasia (HGD), or tubular adenoma larger than 10 mm, as measured by an endoscopist.

### PET/CT technique

PET/CT data were acquired using a Biograph 6 PET/CT scanner (Siemens Medical Solutions; Erlangen, Germany). All patients fasted for at least 6 h before the intravenous injection of 7.4 MBq of 18F-FDG per kg of body weight and the blood glucose level did not exceed 7.2 mmol/L in any of the patients. CT images without intravenous contrast were obtained 60 min after 18F-FDG injections with the following imaging parameters: 130 kVp, 30 mA, 0.6 s per CT rotation, and a pitch of 6. Immediately after the CT acquisition, PET emission data from the vertex to the upper thigh were acquired, with a 16.2-cm axial field of view in 3-dimensional mode at 3.5 min per bed position. The CT data were used for attenuation correction, and the PET images were reconstructed with a conventional iterative algorithm (ordered-subset expectation maximization, two iterations, and eight subsets). Bowel preparation was not required for the PET/CT examination. The standardized uptake value (SUV) was calculated using a standard formula. PET/CT images were reviewed by institutional board-certified nuclear medicine physicians. After review, all patients with any additional abnormal colonic FDG-avid lesions at the proximal colon besides the target CRC lesions were excluded.

### Statistical analysis

Chi-square or Fisher’s exact tests were used to compare categorical variables. For continuous variables, an independent or paired t-test and Wilcoxon rank-sum or signed-rank test were used as required. We used a Kaplan–Meier analysis to estimate the risk of AA/CA detection and the risk of additional colon resection during postoperative follow-up. A log-rank test was performed to compare risk. In cases in which multiple AA/CA was detected during follow-up, the event was recorded when a higher level of AA/CA was detected. The levels of AA/CA were stratified in the following order: carcinoma, HGD, adenoma with villous component, and tubular adenoma ≥ 10 mm.

We used the propensity score matching (PSM) method to minimize baseline differences between the complete and incomplete colonoscopy groups. To calculate the propensity score (PS), which is the estimated probability for an individual patient to be in the incomplete colonoscopy group based on clinical characteristics, we used logistic regression with the following variables: sex, age, stage, and preoperative level of carcinoembryonic antigen (CEA). Then, the individual patients in the incomplete colonoscopy group were matched to the patients with the nearest PS in the complete colonoscopy group. The matching ratio was set to 1:2. For PSM, the R package “MatchIt” (R Foundation for Statistical Computing, Vienna, Austria) was used. The same comparisons undertaken for unmatched patients were performed for PS-matched patients.

All tests were two-sided, and statistical significance was set at *p* < 0.05. Statistical analyses were performed using R software ver. 4.0.2 (R Foundation for Statistical Computing).

## Results

### Baseline pre-surveillance characteristics and surveillance results of unmatched cohort

The baseline characteristics of all 754 selected patients are described in Table 1. The median age of all patients was 62 years (IQR 55–70 years), and the proportion of males was slightly higher. Age and sex distribution were not different in the complete and incomplete groups. However, the stage distribution and CEA levels were significantly different. The incomplete group had a significantly higher CEA level and a more advanced stage. Although the initiation time and total follow-up period of postoperative colonoscopy were significantly different, the median number of postoperative colonoscopies was not different. On postoperative colonoscopy, CA was detected in three patients (one, carcinoma in situ; two, invasive carcinoma), HGD in three, adenoma with villous component in 14, and tubular adenoma ≥ 10 mm in 34 patients. The AA detection frequency in the incomplete colonoscopy group was significantly higher (13.0% vs. 5.5%, *p* = 0.003). However, in the CA group, the difference was not significant (1.4% vs. 0.2%, *p* = 0.16). Among the AA/CA cases, three patients underwent additional colectomy. Two of them underwent surgeries due to invasive carcinoma, and the other patient underwent surgery due to HGD. All other AA/CAs were removed by colonoscopy. The rate of additional surgery did not differ between the groups.

**Table 1:** Descriptive analyses of baseline pre-surveillance characteristics and surveillance results in the unmatched cohort.

	Complete, (N = 616)	Incomplete, (n = 138)	Total (n = 754)	*p* value
**Sex**				0.547
Female	252 (40.9%)	52 (37.7%)	304 (40.3%)	
Male	364 (59.1%)	86 (62.3%)	450 (59.7%)	
**Age, years, median [IQR]**	62.0 [55.0–70.0]	61.0 [52.0–71.0]	62.0 [55.0–70.0]	0.466
**Stage**				< 0.0001
Stage 0	34 (5.5%)	0 (0.0%)	34 (4.5%)	
Stage I	215 (34.9%)	4 (2.9%)	219 (29.0%)	
Stage II	172 (27.9%)	64 (46.4%)	236 (31.3%)	
Stage III	166 (26.9%)	50 (36.2%)	216 (28.6%)	
Stage IV	29 (4.7%)	20 (14.5%)	49 (6.5%)	
**Advanced stage (≥ stage II)**	367 (59.6%)	134 (97.1%)	501 (66.4%)	< 0.0001
**CEA ≥ 7 ng/dl**				< 0.0001
No	510 (83.3%)	81 (59.6%)	591 (79.0%)	
Yes	102 (16.7%)	55 (40.4%)	157 (21.0%)	
**Initiation time of postoperative colonoscopies, months, median [IQR]**	15.0 [12.0–22.0]	10.0 [7.0–16.0]	14.0 [12.0–21.0]	< 0.0001
**Number of postoperative colonoscopies, median [IQR]**	2.0 [ 1.0–2.0]	2.0 [1.0–3.0]	2.0 [1.0–2.0]	0.784
**Follow up period, median [IQR]**	47.0 [20.0–60.0]	36.0 [14.0–57.0]	45.0 [18.0–60.0]	0.002
**Detection of adenocarcinoma**				0.155
No	615 (99.8%)	136 (98.6%)	751 (99.6%)	
Yes	1 (0.2%)	2 (1.4%)	3 (0.4%)	
**Detection of advanced adenoma**				0.003
No	582 (94.5%)	120 (87.0%)	702 (93.1%)	
Yes	34 (5.5%)	18 (13.0%)	52 (6.9%)	
**Pathology**				0.001
None/Tubular adenoma	581 (94.3%)	118 (85.5%)	699 (92.7%)	
Anastomotic recurrence	0 (0.0%)	1 (0.7%)	1 (0.1%)	
Adenocarcinoma	1 (0.2%)	1 (0.7%)	2 (0.3%)	
Adenocarcinoma in situ	0 (0.0%)	1 (0.7%)	1 (0.1%)	
High-grade dysplasia	2 (0.3%)	1 (0.7%)	3 (0.4%)	
Tubular adenoma ≥ 10 mm	25 (4.1%)	9 (6.5%)	34 (4.5%)	
Villous adenoma	7 (1.1%)	7 (5.1%)	14 (1.9%)	
**Additional operation**				1
No	614 (99.7%)	137 (99.3%)	751 (99.6%)	
Yes	2 (0.3%)	1 (0.7%)	3 (0.4%)	

*IQR* interquartile range; *CEA* carcinoembryonic antigen.

### Kaplan–Meier analysis for estimating the AA/CA risk in the unmatched cohort

Kaplan–Meier analysis showed that the cumulative risk of AA/CA detection in all patients was 1.8% at 1 year and 10.1% at 5 years after surgery. However, when evaluated separately, the 1-year cumulative risk for detection of AA was 1.6% and the 5-year cumulative risk was 9.9%, while for CA, detection in all patients was 0.1% at 1 year and 0.4% at 5 years after surgery. Moreover, the risk of additional surgery due to AA/CA in all patients was 0% at 1 year and 0.5% at 5 years after surgery.

**Figure 1:**
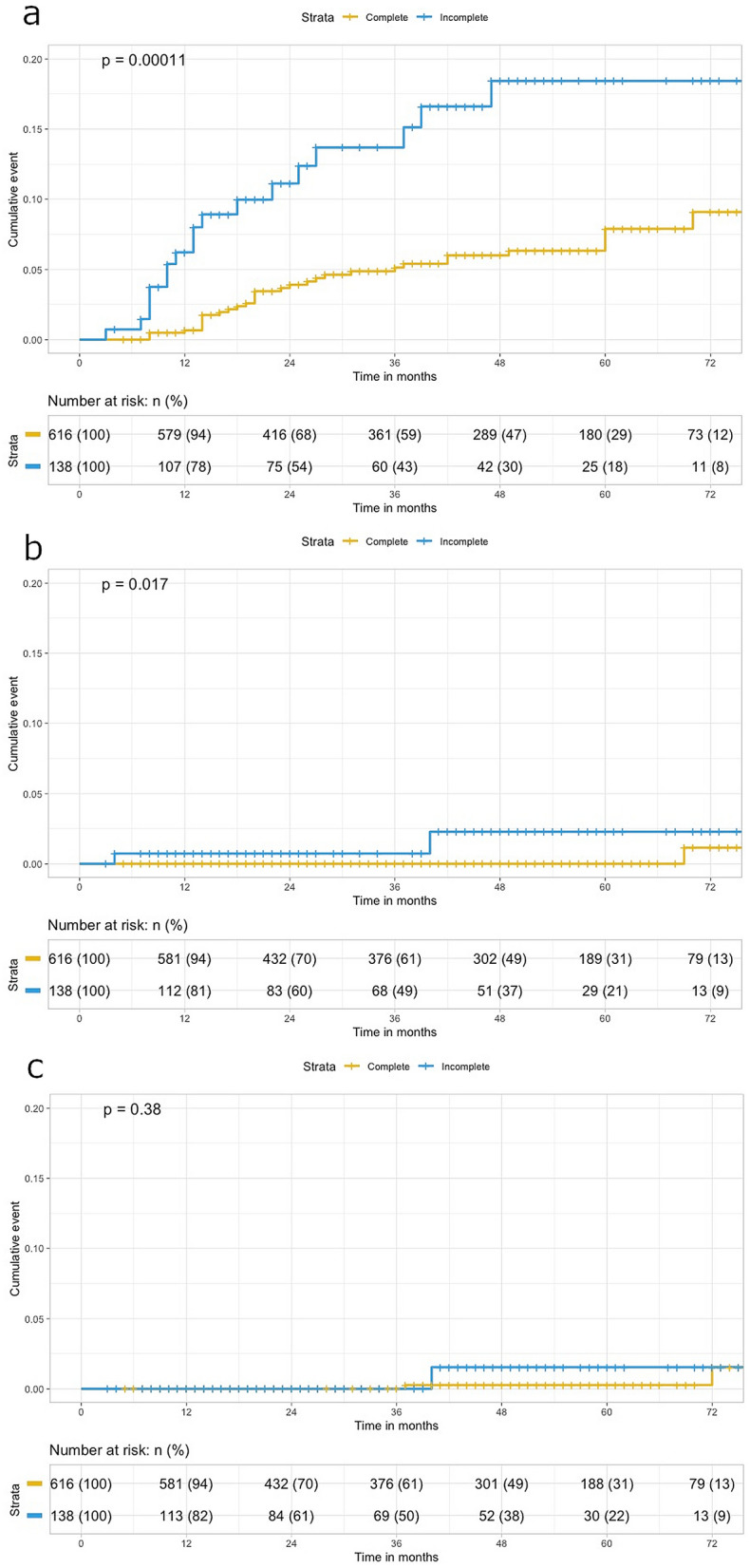
Comparison of cumulative risk after index surgery for detection of advanced adenoma (**a**), carcinoma (**b**), additional operation (**c**) in all enrolled patients.

The log-rank comparison according to the groups is shown in Fig. 1. The cumulative AA detection rate in the complete colonoscopy group was 0.7% at 1 year and 7.9% at 5 years after surgery; however, those of the incomplete group were significantly higher, at 6.2% at 1 year and 12.4% at 5 years (*p* = 0.0001). The rate of CA in the incomplete group was 0.7% at 1 year and 2.3% at 5 years; however, in the complete group, there were no CA cases until 69 months after surgery (*p* = 0.017). Despite this, no patient underwent additional surgery within 3 years in either group, and the cumulative rate of additional surgery at 5 years was 0.3% in the complete group and 1.5% in the incomplete group (*p* = 0.38). During the total follow-up period, three patients underwent additional surgery. In one patient in the incomplete group, invasive cancer was detected during the first colonoscopy at 39 months after the index surgery. The other two patients were in the complete group, and an HGD and invasive cancer were detected during their third surveillance colonoscopy at 37 and 69 months, respectively, after index surgery.

### Analyses in PS-matched patients

**Figure 2:**
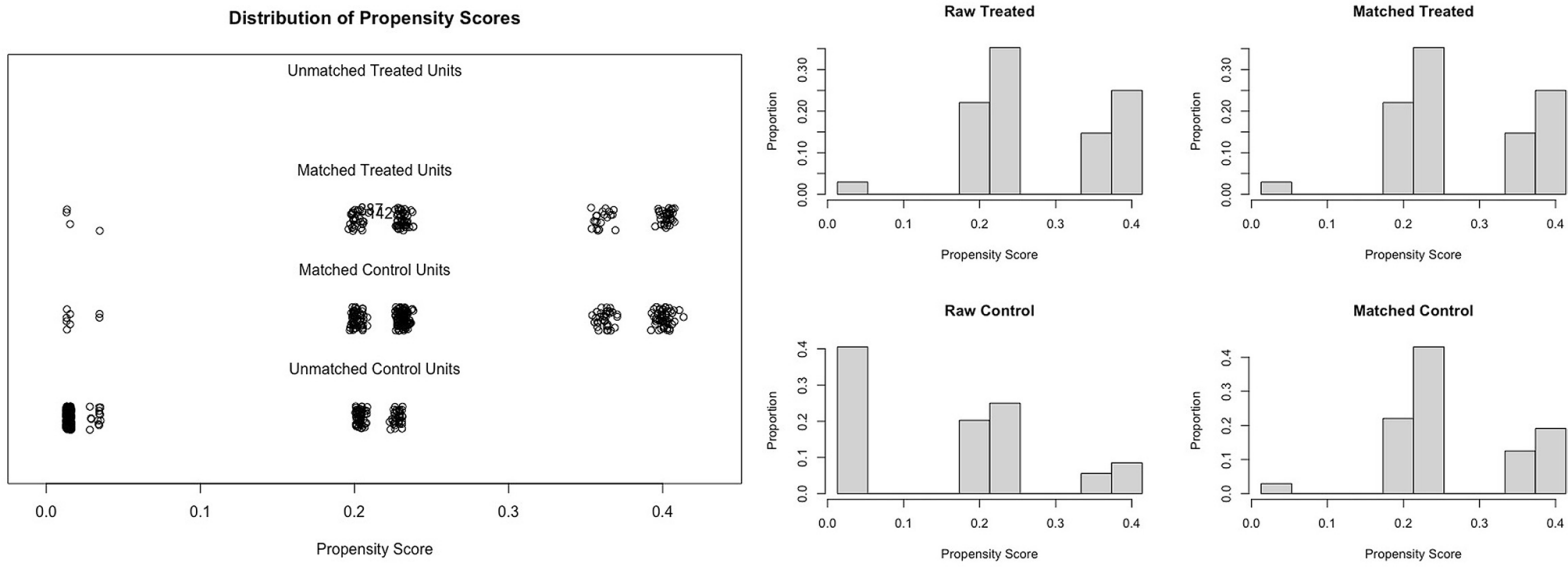
Distribution of propensity score before and after matching.

All patients in the incomplete colonoscopy group (n = 136), except two with missing CEA values, were matched to the 272 patients in the complete colonoscopy group using previously described methods. The imbalances in the baseline pre-surveillance characteristics shown in Table 1 were well-balanced after matching (Fig. 2) (Table 2). In the matched cohort, the frequency of detection of AA/CA was still significantly higher in the incomplete group (*p* = 0.049). However, CA and additional surgery were not different between the groups (*p* = 0.539).

**Table 2:** Descriptive analyses of baseline pre-surveillance characteristics and surveillance results in the matched cohort.

	Complete (N = 272)	Incomplete (n = 136)	Total (n = 408)	*p* value
**Age (years), median [IQR]**	61.0 [55.0–70.5]	61.0 [52.0–71.0]	61.0 [55.0–71.0]	0.68
**Sex**				0.744
Female	98 (36.0%)	52 (38.2%)	150 (36.8%)	
Male	174 (64.0%)	84 (61.8%)	258 (63.2%)	
**Stage**				1
Stage 0/I	8 (2.9%)	4 (2.9%)	12 (2.9%)	
Stage II/III/IV	264 (97.1%)	132 (97.1%)	396 (97.1%)	
**CEA ≥ 7 ng/dl**				0.153
No	183 (67.3%)	81 (59.6%)	264 (64.7%)	
Yes	89 (32.7%)	55 (40.4%)	144 (35.3%)	
**Initiation time of postoperative colonoscopies, months, median [IQR]**	14.0 [12.0–20.0]	10.0 [7.0–15.5]	13.0 [10.0–19.0]	< 0.0001
**Number of postoperative colonoscopies, median [IQR]**	2.0 [1.0–3.0]	2.0 [1.0–3.0]	2.0 [1.0–3.0]	0.853
**Follow-up period**	46.5 [18.5–61.0]	38.0 [14.0–57.5]	43.0 [16.5–60.0]	0.009
**Detection of adenocarcinoma**				0.539
No	271 (99.6%)	134 (98.5%)	405 (99.3%)	
Yes	1 (0.4%)	2 (1.5%)	3 (0.7%)	
**Detection of advanced adenoma**				0.081
No	252 (92.6%)	118 (86.8%)	370 (90.4%)	
Yes	20 (7.4%)	18 (13.2%)	38 (9.6%)	
**Pathology**				0.129
None/Tubular adenoma	252 (92.6%)	116 (85.3%)	368 (90.2%)	
Anastomotic recurrence	0 (0.0%)	1 (0.7%)	1 (0.2%)	
Adenocarcinoma	1 (0.4%)	1 (0.7%)	2 (0.5%)	
Adenocarcinoma in situ	0 (0.0%)	1 (0.7%)	1 (0.2%)	
High-grade dysplasia	2 (0.7%)	1 (0.7%)	3 (0.7%)	
Tubular adenoma ≥ 10 mm	13 (4.8%)	9 (6.6%)	22 (5.4%)	
Villous adenoma	4 (1.5%)	7 (5.1%)	11 (2.7%)	
**Additional operation**				1
No	270 (99.3%)	135 (99.3%)	405 (99.3%)	
Yes	2 (0.7%)	1 (0.7%)	3 (0.7%)	

*IQR* interquartile range; *CEA* carcinoembryonic antigen.

**Figure 3:**
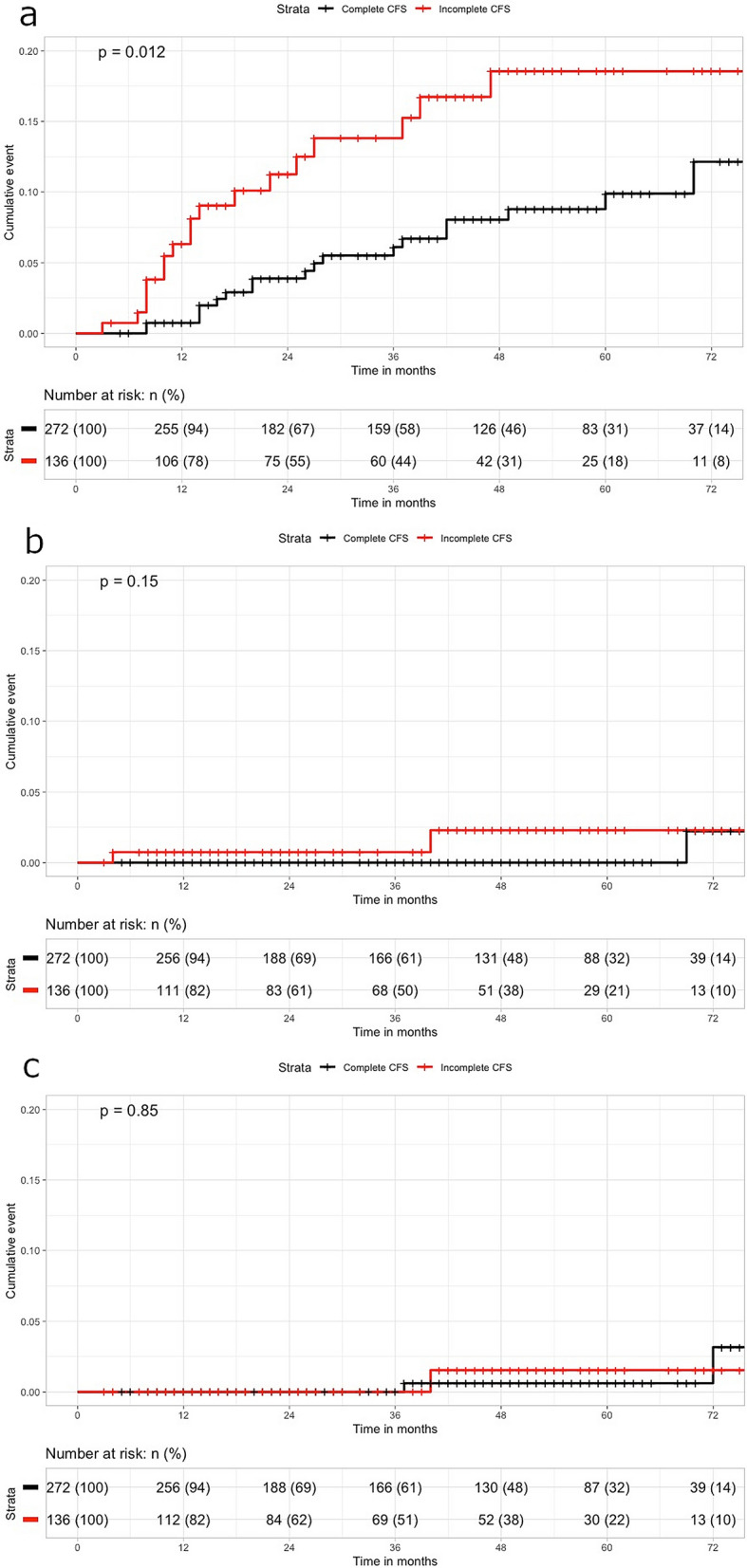
Comparison of cumulative risk after index surgery for detection of advanced adenoma (**a**), carcinoma (**b**), additional operation (**c**) in the matched cohort.

In the Kaplan–Meier analysis for the PS-matched cohort, the cumulative AA detection rate in the incomplete colonoscopy group was still higher than in the complete colonoscopy group (6.3% vs. 0.7% at 1 year, 12.5% vs. 9.9% at 5 years, *p* = 0.012) (Fig. 3). The cumulative rate of detection of CA in the incomplete colonoscopy group was also higher (0.7% vs. 0% at 1 year, 2.3% vs. 0% at 5 years, *p* = 0.15). However, the cumulative rate of additional surgery in the respective groups was not different between groups. The 5-year cumulative rate of additional surgery was 0.6% in the complete group and 1.5% in the incomplete group (*p* = 0.85). As in the unmatched cohort, there were no patients who received additional surgery due to the presence of AA/CA until 3 years after surgery for index CRC.

## Discussion

In this study, even though no enrolled patients had any synchronous FDG-avid lesions at the proximal colon in addition to the surgically targeted CRC, the patients undergoing incomplete preoperative colonoscopy had a significantly higher risk of detection of AA/CA compared with those who underwent complete preoperative colonoscopy, even in the PS-matched cohort. However, the additional surgical risk due to the detected AA/CA was not different between the groups, and the risk was very low.

The long-term follow-up results of our study were concordant with those of previous cross-sectional studies. Kim et al. reported that 18F-FDG PET/CT shows high sensitivity and negative predictive value for detecting proximal synchronous CRC in patients with obstructive CRC. Thus, the negative findings in the proximal colon on PET/CT would enable definite exclusion of proximal synchronous CRC. The results stated that preoperative PET/CT could be recommended to determine the proper surgical plan in patients with obstructive CRC.

However, their recommendations were confined to CRC and not applicable to advanced adenoma. As our results showed, within 12 months, the generally accepted period for defining synchronous CRC, significantly more AA/CA was detected in the incomplete preoperative colonoscopy group. These might be lesions that were missed by both preoperative colonoscopy and PET/CT. The reported sensitivity of PET/CT for CRC is 80–100%; however, it is only approximately 50% for advanced adenoma^11,13,14^. The detectability of PET/CT for adenoma or CRC depends on the size, degree of tumour, and location. According to Igarashi’s report, the detection sensitivity was 40.6% for lesions ≥ 6 mm, 62.5% for lesions ≥ 10 mm, and 85.4% for lesions ≥ 20 mm. In addition, according to tumour grade, the sensitivity of low-grade adenoma was only 9.3% and 50.7% for advanced adenoma, 79.5% for T1 cancer, and 92.9% for advanced cancer. Depending on the colon segment, the detection sensitivity of advanced adenoma was 35% for cecum, 52% for ascending colon, 20% for transverse colon, 54.5% for descending colon, 60% for sigmoid colon, and 63.2% for rectum^14^. Previous studies have reported similar results^15–17^. Considering the result of more detection of advanced adenomas in the incomplete colonoscopy group despite PET negative findings in all subjects of this study, this might be related to the limited sensitivity of PET in the proximal segment of the colon.

Fortunately, the invasive cancer risk was very low, and the cumulative risk of invasive cancer was 0% up to 40 months after the index surgery in our study. The cumulative incidence of metachronous CRC is 0.35% per year^18^. Even considering the incompleteness of the preoperative endoscopy, the cumulative risk of invasive cancer in the incomplete group was almost the same as that of previous studies and was not different from that of the complete group. From these results, preoperative incomplete colonoscopy is not associated with an increased risk of CRC detection and the risk of additional surgery during the synchronous period. However, the low risk of additional surgery over the synchronous period did not mean that the role of postoperative surveillance endoscopy could be overlooked. Colonoscopy surveillance has reduced CRC risk, especially in high-risk patients with a history of CRC^19–23^. Although the risk of AA/CA detection in the incomplete group was significantly higher, even in the matched cohort, the result that the risk of cancer and additional surgery were not different and maintained up to the metachronous period are thought to have been highly influenced by postoperative surveillance colonoscopy.

One concern about the results of our study was that the PET/CT results of no synchronous FDG-avid lesion could be interpreted as a substitute for colonoscopy performed within 3–6 months after surgery as recommended by the current guideline. However, we still believe that the current guidelines should be followed. If there is no synchronous FDG-avid lesion, it may be affordable to delay the timing of postoperative colonoscopy to some extent although it is not possible to say definitively. However, in addition to weaknesses of PET/CT from adenoma’s location and size mentioned before, the sensitivity of PET/CT is dramatically lowered in mucinous cancer and hyperglycaemic patients^24–26^. High-quality perioperative, especially postoperative clearing in the incomplete preoperative colonoscopy group, is very important for detecting missed synchronous colorectal cancer by PET/CT and removing advanced adenoma for preventing future cancer.

The present study had some limitations. First, this was a retrospective analysis which poses a possibility of a selection bias and unmeasured confounders, although efforts were made to reduce the bias by using PSM. The information associated with the quality of preoperative colonoscopies, such as bowel preparation grade, was lacking when patients were referred from outside clinics. In addition, the postoperative surveillance schedule was not standardized among surgeons, and the initiation of surveillance colonoscopy was not uniform despite guidelines^3^. Second, this was a single-centre study, and the small sample size, which was due to the low incidence of synchronous CRC, might reduce the statistical power of the results. Third, PET/CT is an easily accessible modality under the coverage of the National Health Insurance System for initial staging in Korea. However, this may not be the case in other countries because of cost issues. Therefore, the results of this study should be interpreted carefully and may not be generalised.

In conclusion, in patients undergoing preoperative incomplete colonoscopy, the finding of negative incidental PET lesions in the proximal colon other than the target CRC ensures that there are no additional lesions that warrant a change in the surgical plan.
